# Cyanobacteria blooms induced precipitation of calcium carbonate and dissolution of silica in a subtropical lagoon, Florida Bay, USA

**DOI:** 10.1038/s41598-023-30905-4

**Published:** 2023-03-11

**Authors:** Jia-Zhong Zhang

**Affiliations:** Ocean Chemistry and Ecosystems Division, Atlantic Oceanographic and Meteorological Laboratory, National Oceanic and Atmospheric Administration, Miami, FL 33149 USA

**Keywords:** Biogeochemistry, Environmental sciences, Ocean sciences

## Abstract

In recent decades, annual cyanobacteria blooms in Florida Bay displayed spatial and temporal patterns that are consistent with changes in alkalinity and dissolved silicon in water. In early summer, the blooms developed in the north–central bay and spread southward in fall. The blooms drew down dissolved inorganic carbon and increased water pH, causing in situ precipitation of calcium carbonate. Dissolved silicon concentrations in these waters were at minimum in spring (20–60 µM), increased during summer, and reached an annual maximum (100–200 µM) during late summer. The dissolution of silica as a result of high pH in bloom water was first observed in this study. During the peak of blooms, silica dissolution in Florida Bay varied from 0.9 × 10^7^ to 6.9 × 10^7^ mol per month over the study period, depending on the extent of cyanobacteria blooms in a given year. Concurrent calcium carbonate precipitations in the cyanobacteria bloom region are between 0.9 × 10^8^ and 2.6 × 10^8^ mol per month. It is estimated that 30–70% of atmospheric CO_2_ uptake in bloom waters was precipitated as calcium carbonate mineral and remainders of CO_2_ influx were used for the production of biomass.

## Introduction

In recent decades, cyanobacteria blooms have occurred worldwide spanning from freshwater lakes and rivers to estuarine waters and coastal lagoons^1–5^. Frequent cyanobacteria blooms around the world have attracted scientific and public attention such as the Baltic Sea in northern Europe, Bolmon lagoon in south France, the Caspian Sea in west Asia, Lake Victoria in Africa, Lake Erie in North America, Lake Taihu in China, and Lake Kasumigaura in Japan^6–12^. The proliferation and expansion of cyanobacteria often forms harmful algal blooms that produce toxins, alter the food web, and generate hypoxia in aquatic ecosystems^6–12^. They not only affect ecosystem health but also represent a serious threat to the availability and sustainability of our freshwater and brackish water resources^13–15^. Many studies have focused on the environmental conditions that trigger cyanobacteria blooms, including eutrophication, nutrient loading, and high water temperature^16–26^. Recent studies demonstrated that cyanobacteria bloom not only uptake CO_2_ from the air but also release another greenhouse gas, methane, to the air^27^.

**Figure 1:**
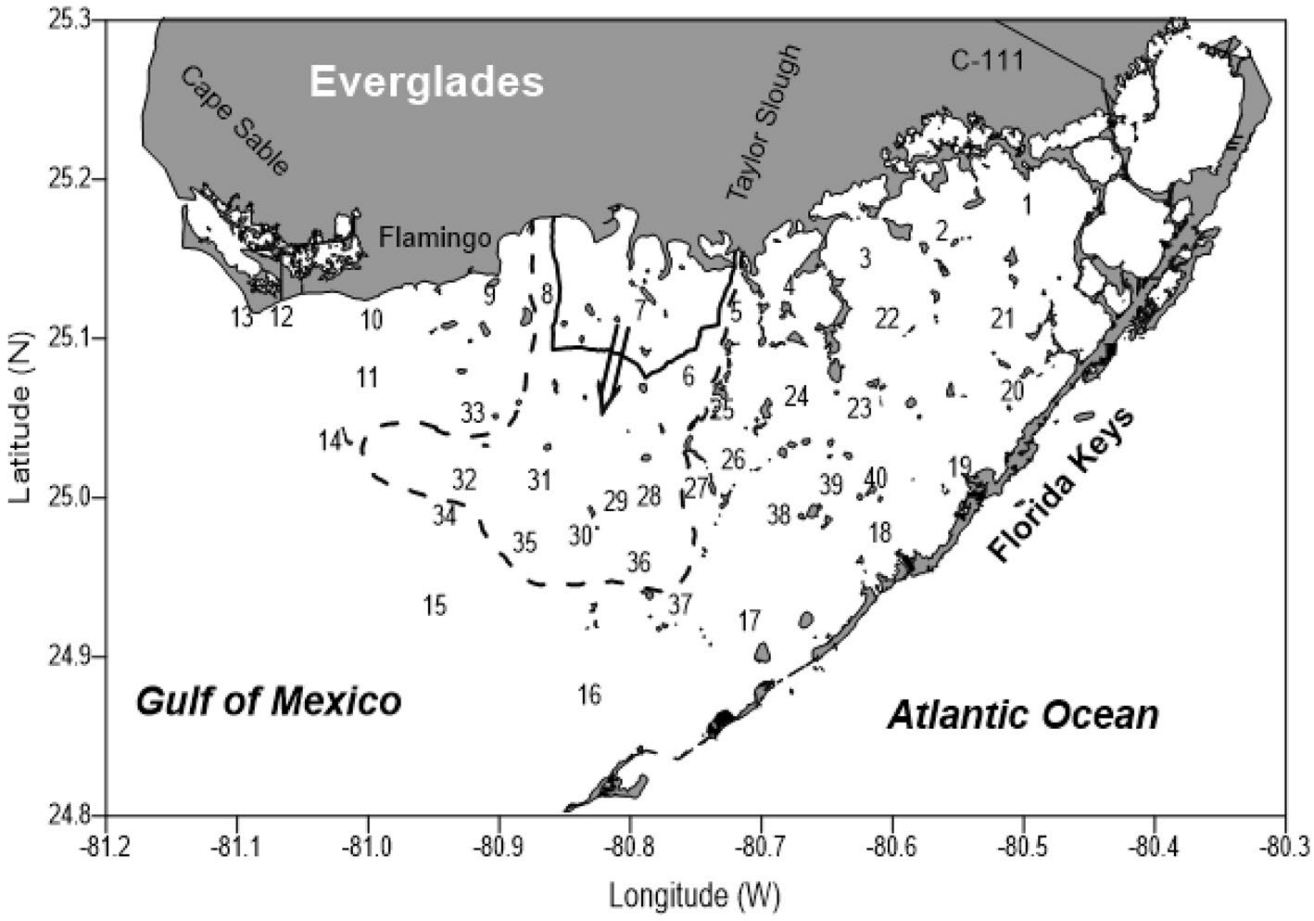
Locations of 40 sampling stations in Florida Bay. Seasonal progression of cyanobacteria bloom is indicated by the arrow and contour lines: summer in solid line and fall in dash line (after Phlips et al.^29^).

Florida Bay is located at the southern end of the Florida peninsula, USA and separated from the Atlantic Ocean by a 180-mile island chain called the Florida Keys. It is one of the largest coastal lagoons in the world with an area of approximately 2000 km^2^. Since 1991, Florida Bay has experienced persistent cyanobacteria blooms. The cyanobacteria blooms in Florida Bay have displayed similar spatial and temporal patterns every year^28–31^. Many studies documented the development of annual blooms of cyanobacteria in Florida Bay in late spring or early summer. It has been demonstrated that the north-central bay experienced high frequency and intensity of the blooms with cellular biovolumes of the cyanobacteria *Synechococcus* regularly exceeding 10^7^ µm^3^ ml^−1^ (Fig. 1)^29^. High concentration of cyanobacteria spread to the south-central bay during the fall. This transport pattern coincided with the onset of seasonal cold fronts in fall. During fall season, the cold front brought a strong northerly or northwesterly wind that can drive the bloom-laden water from the north-central bay to the south-central Bay^29^. The observed similar patterns of salinity variation in these two regions supported the seasonal wind as a driving force in water circulation in the central region of Florida Bay^32^.

Cyanobacteria process two modes of CO_2_ capture: photosynthesis convers CO_2_ to organic carbon and calcification combines calcium ion with CO_2_ to calcium carbonate minerals. The widely accepted mechanism in calcification involves the carbon dioxide concentrating mechanism^33^. Cyanobacteria cells are able to raise the concentration of CO_2_ at the site of the carboxylating enzyme ribulose 1,5-bisphosphate carboxylase/oxygenase (Rubisco) up to 1000-fold over that in the ambient waters, elevating pH at cell exterior and thus facilitating calcium carbonate precipitation^34^. The nucleation of calcite on the sheaths of cyanobacteria has been observed in previous study^35^.

In calcification process, every mole of calcium carbonate mineral formation consumes two moles of alkalinity as follows.

$${\text{Ca}}^{{{2} + }} + {\text{2HCO}}_{{3}}^{ - } = {\text{CaCO}}_{{3}} + {\text{CO}}_{{2}} + {\text{H}}_{{2}} {\text{O}}$$

The photosynthesis by cyanobacteria in bloom waters draw down dissolved inorganic carbon and raise the pH. The rising pH in shallow bay water has resulted in precipitation of calcium carbonate and dissolution of silica in sediment.

Dissolution of silica in water at circumneutral pH is initiated by nucleophilic attack of water molecules that cause the breaking of siloxane bonds, > Si–O–Si < , at the particle surface^36^. As a water molecule approaches a surface silicon atom, the transfer of electron density weakens and eventually breaks the adjacent siloxane Si–O bond and bind with the dissociating water molecule to form Si–OH (silanol) groups. The process is then repeated until all siloxane bonds surrounding a surface silicon atom are broken and the formation of dissolved silicic acid molecule, Si(OH)_4_. A charged hydroxide ion is a stronger nucleophile than molecular water because it leads to the deprotonation of surface silanol groups, thereby further facilitating the breaking of the bridging siloxane bonds. Base-promoted dissolution of silica has been demonstrated previously for marine diatom test and plant phytoliths^37^.

On the other hand, both freshwater and seawater are under-saturated with respect to solubility of most silica minerals, which is about 2 mM. Dissolution of silica is thermodynamically favorable in most natural waters but dissolution rate is kinetically limited. The dissolution rate is enhanced by higher pH as evidenced by a study that the rate increased five times from pH 6.3 freshwater to pH 8.1 seawater^38^. Our previous study has documented that cyanobacteria blooms have taken-up the atmospheric CO_2_ and bloom waters act as a CO_2_ sink in Florida Bay^39^. The objective of this study is to quantify the annual budget of calcium carbonate precipitation and silica dissolution induced by cyanobacteria blooms in Florida Bay.

## Methods

### Sampling and analysis

Water samples for nutrient analysis were collected from 40 stations within Florida Bay on the bimonthly survey aboard the R/V *Virginia Key* from 1999 to 2012. Water pH and dissolved inorganic carbon measurements were added after 2006. Detail information on sampling and sample analysis has been published elsewhere^39^. Briefly, the filtered seawater samples were analyzed at our shore-based laboratory for dissolved inorganic carbon, pH, and nutrients. Dissolved inorganic carbon was analyzed using a Shimadzu TOC-V total organic carbon analyzer in inorganic carbon mode. Seawater samples were acidified with hydrochloric acid to release carbon dioxide, and the latter was detected by a non-dispersive infrared gas analyzer. The pH of seawater sample was measured by a spectrophotometric method using m-cresol purple as an indicator dye. Absorbance changes were quantified by an Agilan-8453 spectrophotometer at wavelengths of 578 and 434 nm^40^. Values for pH were then calculated in total proton scale at 25 °C using indicator dye pK values that were measured over the estuarine salinity range^41^.

Nutrient samples were analyzed with a Seal autoanalyzer for nitrate, nitrite, phosphate and silicate concentrations. Nitrate in the samples was first reduced to nitrite by a copper-coated cadmium column^42^, and the resulting nitrite concentration (i.e., the sum of nitrate + original nitrite) was then determined by diazotization reaction with sulfanilamide and coupling with N-1 naphthyl ethylenediamine dihydrochloride to form a pink azo dye, the absorbance of which was measured at 540 nm^43^. Phosphate in the sample was determined by its reaction with molybdate acid to form a phosphomolybate complex that was subsequently reduced with hydrazine to form phosphomolybdium blue, the absorbance of which was measured at 736 nm^44^. Silicate was analyzed by its reaction with ammonium molybdate in an acidic solution to form β-molybdosilicic acid, which was then reduced by ascorbic acid to form molybdenum blue. The absorbance of the molybdenum blue was measured at 660 nm. Oxalic acid was added to prevent reduction of excess molybdate and to minimize interference of phosphate in the sample^45^.

### Calculation of inorganic carbon parameters and inventory changes in alkalinity and dissolved silicon

The inorganic carbon parameters in seawater were calculated using CO2SYS.XLS from measured dissolved inorganic carbon concentration, pH, salinity, temperature, nutrients, and the carbonic acid dissociation constants in the total proton scale^46–48^. Detail procedures for calculation of inorganic carbon parameters have been published elsewhere^39^. Briefly, the carbonate alkalinity and calcium carbonate saturation state (Ω) in the seawater samples was calculated from measured values of pH and dissolved inorganic carbon concentration. By definition of carbonate alkalinity^49^, biological processes involving CO_2_, such as photosynthesis and respiration, do not change the carbonate alkalinity of ambient water, any change in carbonate alkalinity is attributed to precipitation or dissolution of calcium carbonate minerals. However, total alkalinity does change slightly in photosynthesis or respiration processes as a result of assimilation or release of inorganic nutrient species^50^.

Bay-wide dissolved silicon and carbonate alkalinity for each survey was integrated from the 40 stations using contour grid files created with Surfer software. Surfer is a grid-based mapping program that interpolates irregularly spaced survey station data into a regularly spaced grid. The grid is used to produce different types of maps including contour and 3D surface maps. The bay-wide inventory of dissolved silicon and carbonate alkalinity for each survey was calculated using the grid volume computation function in Surfer with input of average water depth in Florida Bay.

## Results

### Spatial distributions of total alkalinity, calcium carbonate saturation index (Ω), pH, and dissolved silicon concentration and their seasonal variation

**Figure 2:**
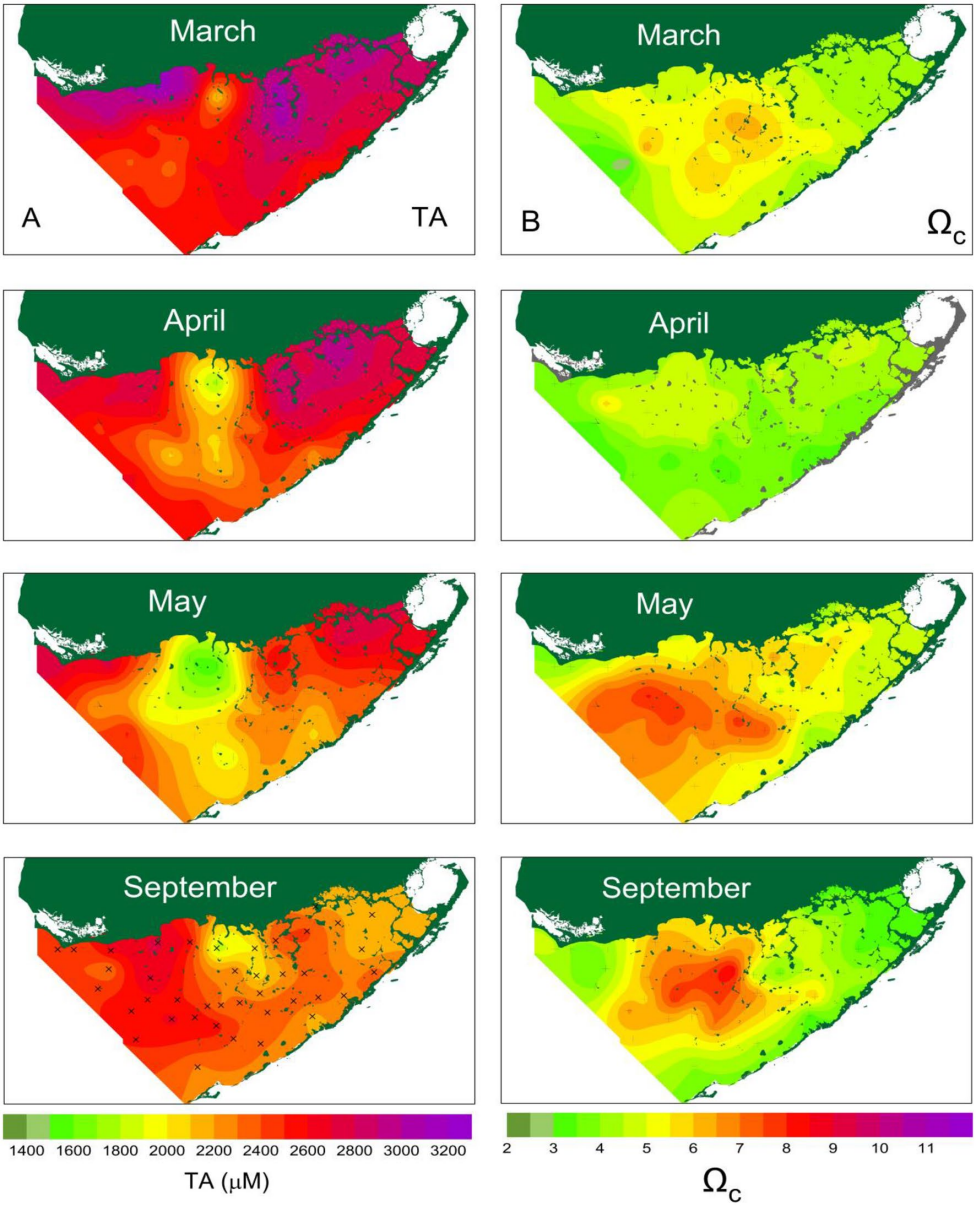
Seasonal changes in spatial distribution of (**A**) total Alkalinity (TA,1400–3200 µM, interval 200 µM) and (**B**) saturate state Ω_c_ (2–11, interval 1) in Florida Bay waters during year 2011. The contour maps were generated using a commercial software Surfer version10 (https://www.goldensoftware.com).

In this study, total alkalinity and calcium carbonate saturation state (Ω) were calculated from measured dissolved inorganic carbon, pH, salinity and nutrients. As example, the bay wide spatial distributions of total alkalinity and Ω and their seasonal variation in 2011 are shown in Fig. 2.

In March, total alkalinity (TA) was high over the entire bay, averaging 2720 ± 221 µM; Ω were moderate, ranging from 3.13 to 5.85 with an average of 4.99 ± 0.76. The highest TA was found in the northern coastal stations adjacent to the Everglades wetlands, ranging from 2801 to 3154 µM with an exception of the lowest TA (2058 µM) and highest pH (8.10) at north-central station 7.

In April, bay wide TA decreased to 2502 ± 284 µM. This was primarily due to a decrease of TA at station 7 (2058 µM) and the spreading of low TA (< 2100 µM) waters southward to the central bay. By the end of May, low TA and high pH waters reached its maximum extent in the central bay, spreading from the coastal waters off the Everglades in the north to the waters close to the Florida Keys in the south. However, the lowest TA of 1544 µM and the highest pH of 8.53 remained at the station 7. The Ω also reached annual maxima (8.15 at station 33 in the northcentral bay) and a largest spatial extent, spreading over entire central bay.

As the season progressed into fall, the area of low TA and high Ω water decreased significantly, but the station 7 remained the lowest TA (1811 µM) and the second highest pH (8.22) in the bay. The high Ω water was observed in the central bay. In October, TA in the eastern bay increased to above 2500 µM, but the low TA waters of the central bay had continued to spread further south. By December, TA increased over the entire bay and the low TA water seen previously had completely disappeared.

The spatial distribution and seasonal variation of TA was opposite to that of salinity in the bay. The highest TA observed in the northeast corner in winter were associated with the lowest salinity (~ 18) in the bay^32,51^. In the early summer, the highest salinity (> 40) developed in the north central bay coexisted with the lowest TA observed^32,51^.

**Figure 3:**
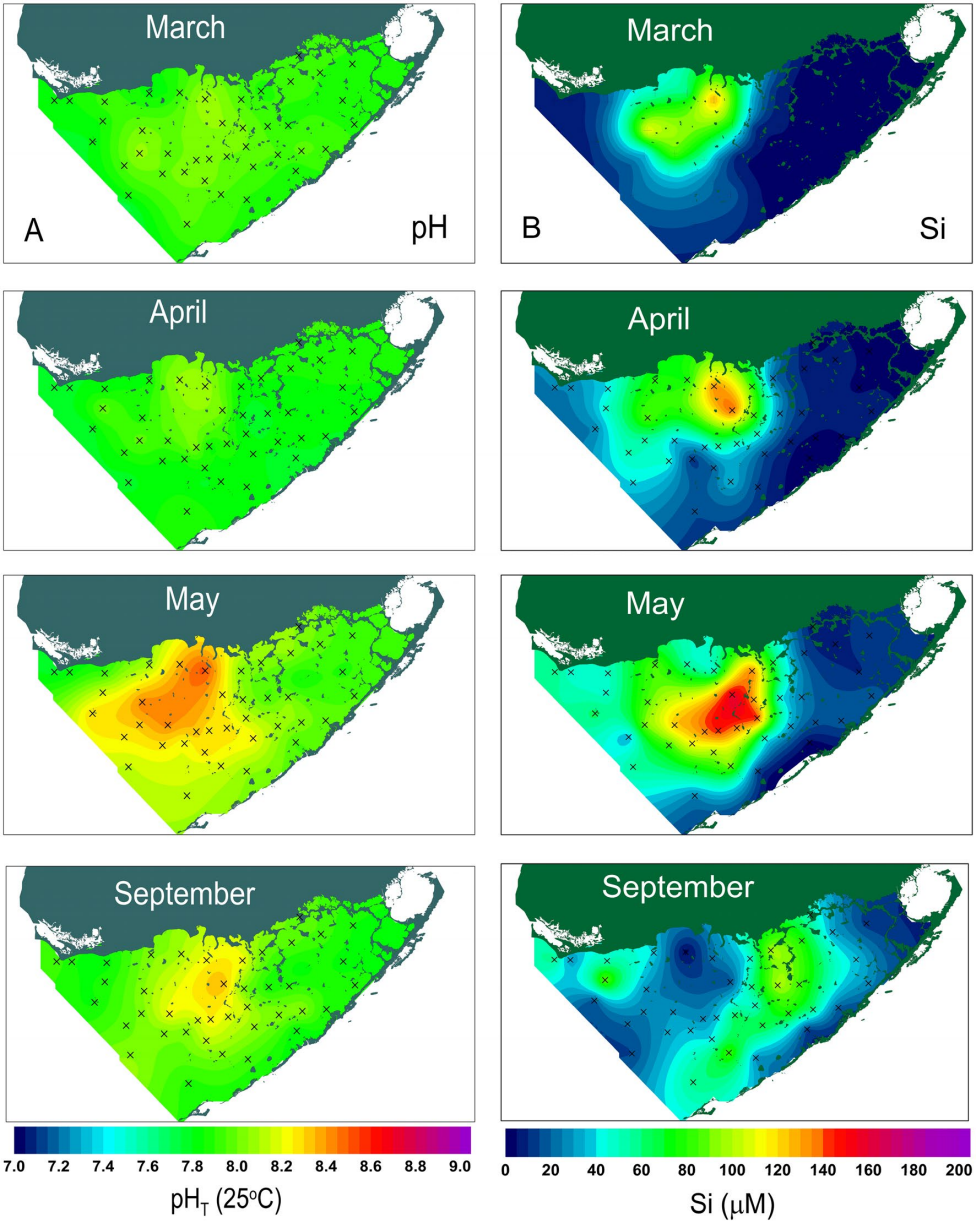
Seasonal changes in spatial distribution of (**A**) pH (7.0–9.0, interval 0.2) and (**B**) dissolved silicon (Si, 0–200 µM, interval 20 µM) in Florida Bay waters during year 2011. The contour maps were generated using a commercial software Surfer version10 (https://www.goldensoftware.com).

The spatial distributions of pH and dissolved silicon concentration and their seasonal variation in 2011 are shown in Fig. 3. The spatial distribution of pH was opposite to that of DIC and TA as a result of carbonate buffering in seawater pH^52,53^. In March, pH was at annual minima, ranging from 7.69 to 8.10 with an average of 7.93 ± 0.08. pH reached annual maxima in May and decreased in September.

High dissolved silicon concentrations were observed in cyanobacteria bloom waters (Fig. 3). In March, dissolved silicon concentrations were low over the entire bay with exception of north central region where maximum dissolved silicon reached 130.6 µM at the station 7. In April, dissolved silicon concentration increased to 133.8 µM but maxima dissolved silicon (142.8 µM) was found at a nearby station 6. By the end of May, high dissolved silicon water reached its maximum extent in the central bay with maxima concentration of 159.2 µM remained at the station 6 but high  dissolved silicon water was spreading south and west directions. As the season progressed into fall, the dissolved silicon concentration decreased significantly and the high dissolved silicon water seen in summer had completely disappeared.

**Figure 4:**
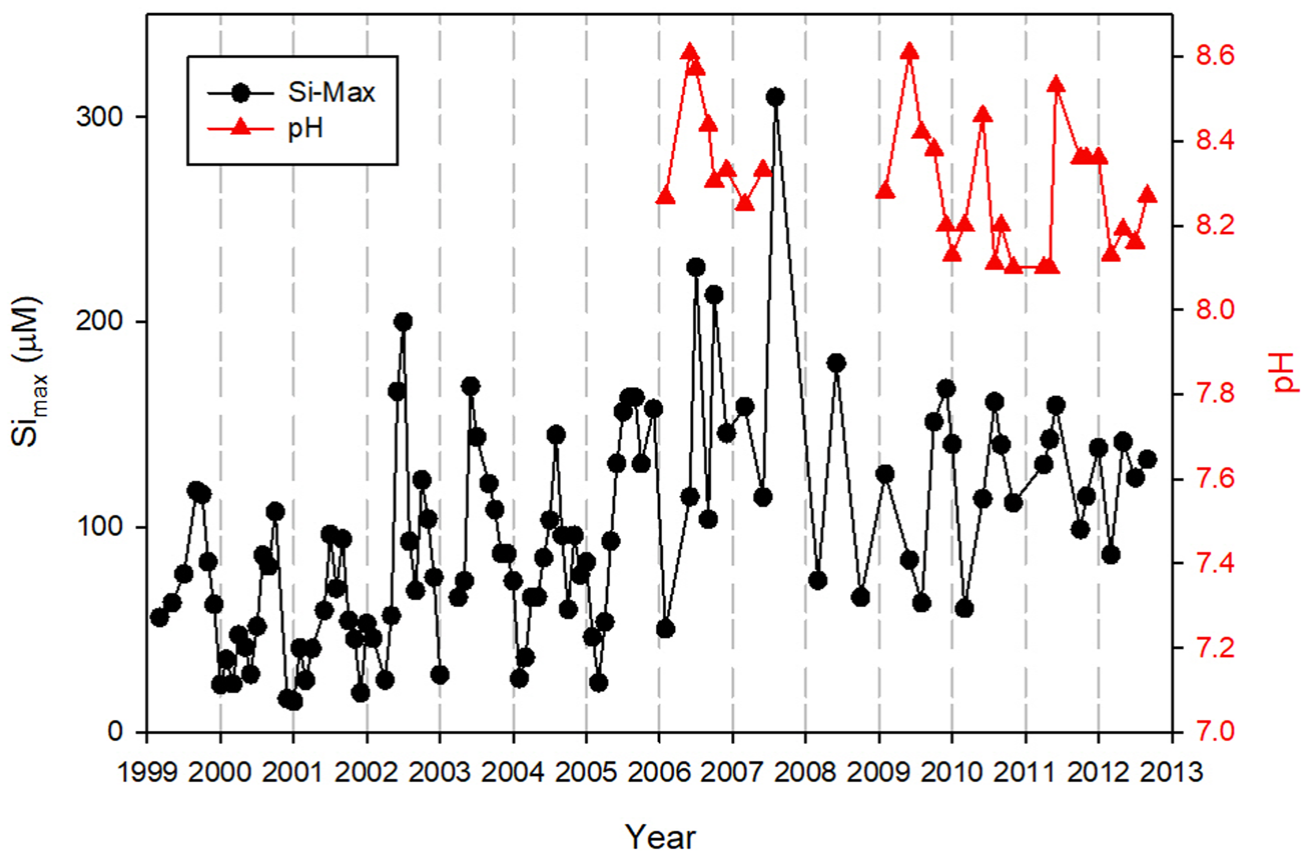
Seasonal and inter-annual changes in maximum dissolved silicon concentration and the monthly maximum pH observed in Florida Bay over the period of 1999–2012.

As shown in 2011 data the maximum pH and dissolved silicon concentrations are always occurred at the center of cyanobacteria blooms in the bay. The monthly maximum dissolved silicon concentrations observed in the bay from 1999 to 2012 are plotted together with observed pH maxima from 2006 to 2012 in Fig. 4. Maxima dissolved silicon concentrations are low in winter and reach peak values every summer. The highest dissolved silicon concentration observed in Florida Bay was 300 µM in summer of 2007. During winter seasons, dissolved silicon concentrations were below 20 µM before 2005. After high dissolved silicon input in 2007 summer, winter dissolved silicon increased to above 50 µM and reached to 100 µM in 2011 and 2012 in Florida Bay. The measured pH was associated with maximum dissolved silicon and varied from 8.1 to 8.6. In spring, the maximum pH was 8.2 ± 0.1 and rapidly increased to 8.5 ± 0.1 in May. Maxima pH decreased gradually in fall and winter to 8.3 ± 0.1.

### Bay-wide integrated monthly inventory of silica dissolution and calcium carbonate precipitation and their interannual variation

**Figure 5:**
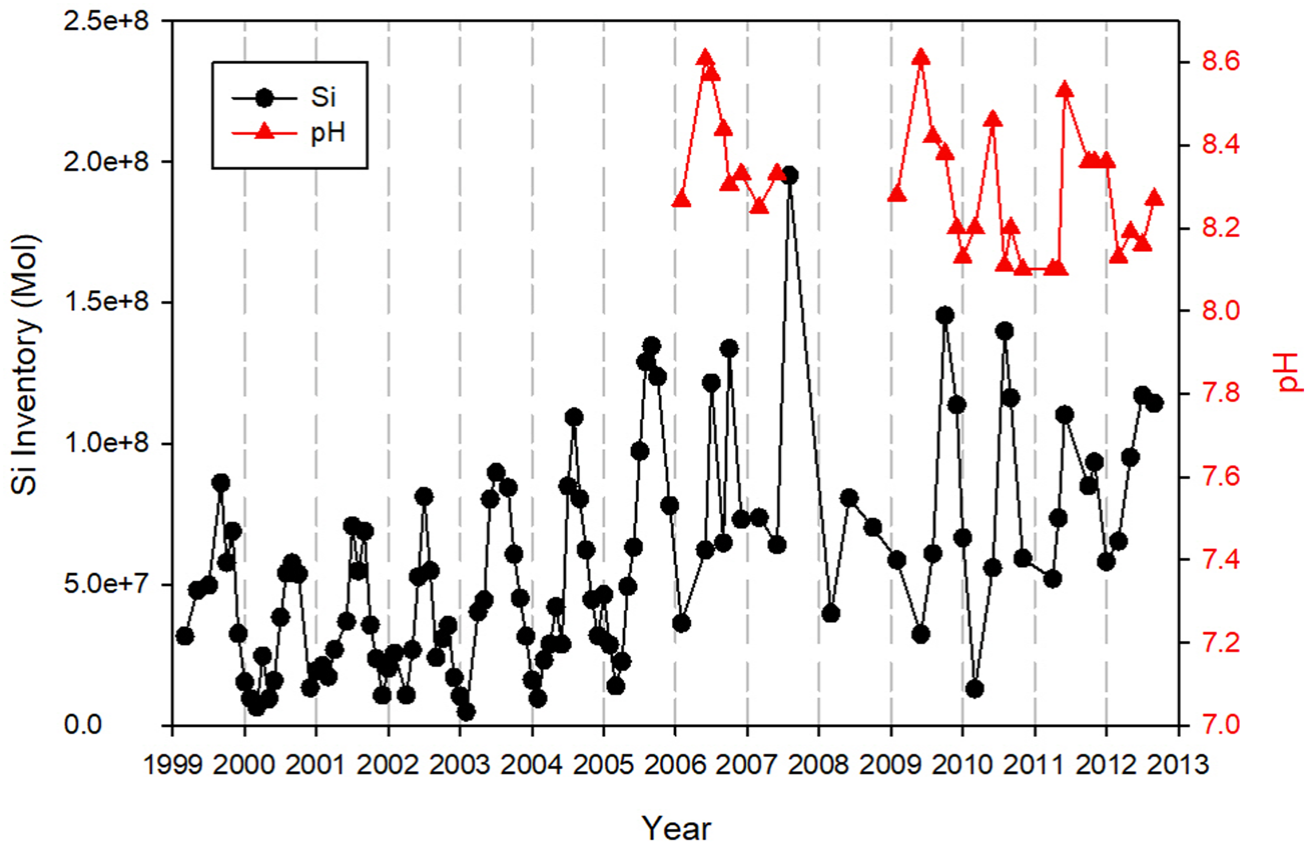
Seasonal and inter-annual changes in maximum pH and inventory of dissolved silicon in Florida Bay over the period of 1999–2012.

The seasonal and interannual variation in the bay-wide dissolved silicon inventory is shown in Fig. 5, which is similar to maximum dissolved silicon concentration because the inventory is dominated by high-dissolved silicon waters. Bay-wide dissolved silicon inventory varied from 1.0 × 10^7^ to 2.0 × 10^8^ mol with an average of 1.0 × 10^8^ ± 1.25 × 10^7^ mol over the 1999–2012 period. The minimum bay-wide dissolved silicon inventory of 1.0 × 10^7^ mol was observed in winter of 1999–2005. The winter minima increased to above 7.0 × 10^7^ mol during 2006 and 2007. It decreased to 1.0 × 10^7^ mol in 2010 but increased again thereafter. The annual maxima of bay-wide dissolved silicon inventory occurred in May due to cyanobacteria blooms. A substantial interannual variation was observed on top of the seasonal pattern.

**Figure 6:**
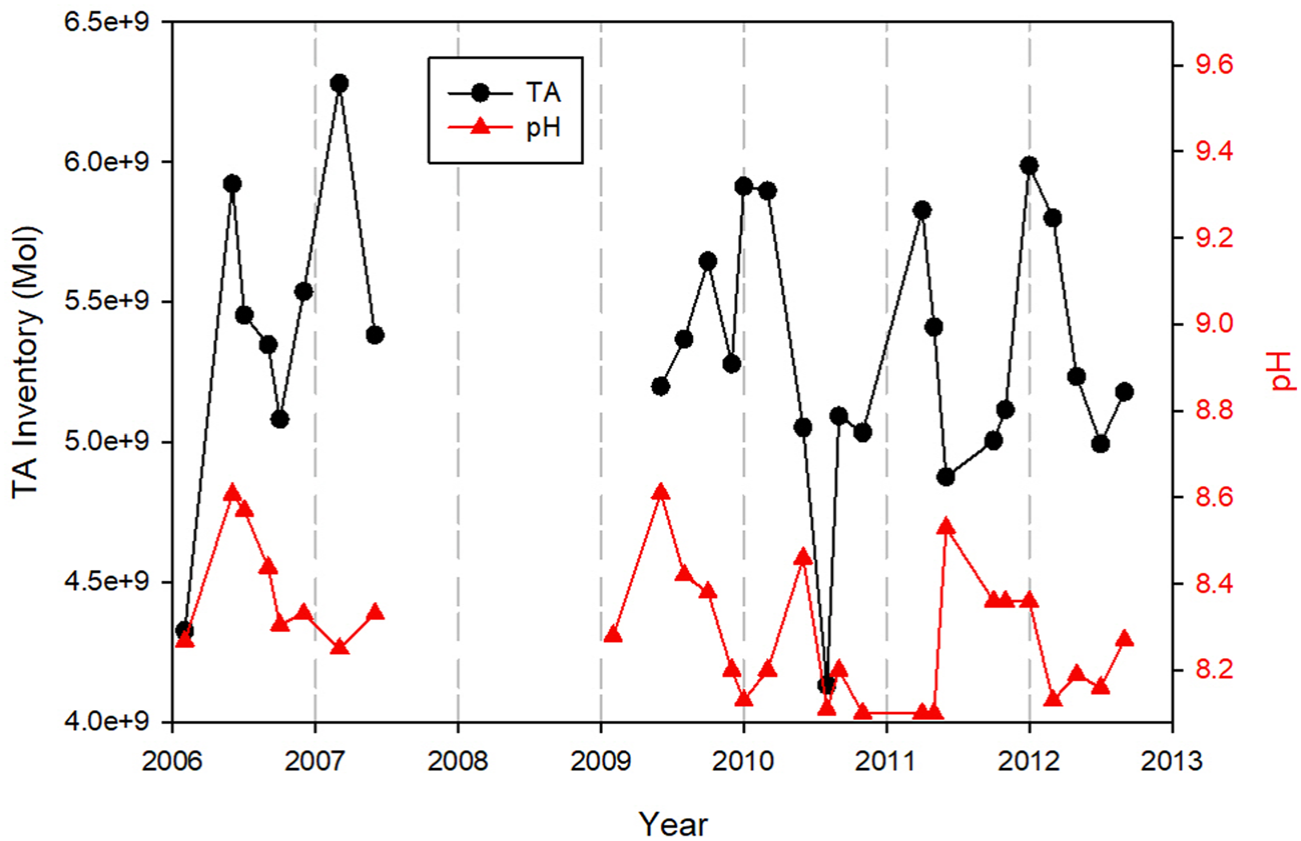
Seasonal and inter-annual changes in maximum pH and inventory of total alkalinity in Florida Bay over the period of 2006–2012.

The annual maxima of bay-wide dissolved silicon inventory varied from a low of 9.0 × 10^7^ ± 1.0 × 10^7^ mol in 1999–2004 to a high of 2.0 × 10^8^ mol in 2007. Concurrent calcium carbonate precipitations in the cyanobacteria bloom region are between 0.9 × 10^8^ to 2.6 × 10^8^ mol per month (Fig. 6). It is estimated that 30–70% of atmospheric CO_2_ uptake in bloom waters ended up as calcium carbonate precipitation and the remaining CO_2_ influx was used for the production of biomass.

## Discussion

As shown in Figs. 2 and 3, bay wide alkalinity decreases from north to south. This is a result of northern Florida Bay receiving substantial material input as runoff from Everglades wetland. The high alkalinity in the northern bay was produced from dissolution of calcium carbonate-rich sediment by the acid generated from respiration of organic matter, which are produced in situ from coastal mangrove forest and runoff from Everglades wetlands into the northern bay^54^. The cyanobacteria bloom developed in north central bay induced calcium carbonate precipitation and lower the alkalinity in bloom region. Overall, the substantial changes in bay-wide total alkalinity are driven by water column and benthic calcification (decrease in alkalinity) and organic matter oxidation (increase in alkalinity). This is in contrast to open ocean waters, where one usually can observe small co-variation of salinity and total alkalinity by freshwater input or loss via evaporation processes.

The observed accumulation of high dissolved silicon in Florida Bay is partly due to the shallow water of the bay (average depth 1 m) where resuspension of sediments is likely to increase the contact of silica minerals, including biogenic silica, with high pH water^38^. To our best knowledge this is the first field observation of dissolution of silica from sediments in high pH bloom water. This is in part due to many coastal water quality studies focusing only on nitrogen and phosphorus and not measuring the dissolved silicon concentration. In deep-water aquatic ecosystems such as ocean or lakes, however, the bloom induced high pH surface waters have little chance in contact with bottom sediment to cause dissolution of silica minerals and therefore it might have not been able to observe accumulation of dissolved silicon in the water column.

Precambrian fossil stromatolites provide evidence of the long evolutionary history of marine cyanobacteria^55,56^. More than three billion years of evolution has provided them with physiological adaptations to extreme environmental conditions and mechanisms to take up and store essential nutrient elements intracellularly^56,57^. Noteworthy abilities include surviving at extreme temperatures from ice-covered to hot spring habitats. Many field observation and culture experiments have shown cyanobacteria are able to precipitate calcium carbonate from ambient waters^35,58,59^, and different forms of calcium carbonates might be formed from different environments, such as aragonite precipitated in seawater, high-magnesium calcite in brackish water, and low-magnesium calcite in fresh water^60,61^. However, a number of studies have also shown dissolution of silica and iron oxides minerals by cyanobacterial communities^62,63^. In this study we provided the first field observational evidence of bay-wide precipitation of calcium carbonate and dissolution of silica by the annual cyanobacteria blooms in one of world’s largest coastal lagoons, Florida Bay, USA.

Although the annual cyanobacterial blooms have been documented in Florida Bay only recent decades, blooms might occur over much longer timescales. Precipitation of calcium carbonate in the western bay is mostly in the form of high-magnesium calcite^54^. The average water depth is about one meter in the Florida Bay, but the western region of the bay is shallower with extended calcium carbonate mudbanks^32^. Continuous accumulation of calcium carbonate derived from the in situ cyanobacterial blooms might increase the sediment production rate and therefore raise the bay floor and reduce the water depth in the western region of the bay.

Generally, the dissolved silicon content is low in tropical and subtropical rivers^64^. Indeed, Shark River in the subtropical Everglades is low in dissolved silicon. However, dissolution of biogenic silica have occurred at land–ocean transition zone around the globe^65^. Such dissolution is enhanced by higher pH and salinity of seawater^38^. Diatom blooms also occur frequently in the western bay^28,66^. The cyanobacteria blooms in the region might provide sufficient dissolved silicon in high pH water, together with high concentration of equilibrium phosphate from sediment in the region^67^, to initiate and sustain the diatom bloom in the western bay. Overall, the cyanobacteria blooms have driven nutrients and carbon dynamics and affected the ecosystem function of the Florida Bay.

## Concluding remarks

This study documented annual cyanobacteria blooms induced substantial precipitation of calcium carbonate and dissolution of silica in Florida Bay. Blooms induced monthly calcium carbonate precipitations in a range of 0.9 × 10^8^ to 2.6 × 10^8^ mol and monthly dissolution of silica in a range of 0.9 × 10^7^ to 6.9 × 10^7^ mol. The bloom waters are CO_2_ sink in Florida Bay and estimated 30–70% of atmospheric CO_2_ uptake in bloom waters was precipitated as calcium carbonate mineral and remainders of CO_2_ influx were used for the production of biomass.

Future study in Florida Bay needs to annually monitoring cyanobacteria blooms in order to identify the environmental factors that regulate the intensity of blooms. In addition to quantify CO_2_ flux in Florida Bay it also needs to examine the potential source of methane from cyanobacteria bloom waters to close the carbon budget of the bay.

## Data Availability

The data used in this paper are available on NOAA Atlantic and Meteorological Laboratory website: https://www.aoml.noaa.gov/data-products/#so-flo-cruise-data.
